# Circulating miRNA-21 as early potential diagnostic biomarker for acute myocardial infarction: a meta-analysis

**DOI:** 10.3389/fcvm.2024.1330884

**Published:** 2024-08-22

**Authors:** Ke Wang, Kai Li, Zhuoyuan Li, Xizhang Yan

**Affiliations:** ^1^ Department of Clinical Medicine, Xi’an Medical University, Xi’an, Shannxi, China; ^2^ Department of the Project of Prevention and Treatment of Respiratory Diseases, Xi’an Medical University, Xi’an, Shannxi, China; ^3^ Department of Emergency, Hanjiang Hospital Affiliated to Xi'an Medical University, Hanzhong, Shannxi, China

**Keywords:** AMI, promising biomarker, miRNA-21, meta-analysis, miR-21

## Abstract

**Introduction:**

There exists a knowledge gap concerning the clinical significance of miRNA-21; therefore, in the present study, we aimed to estimate the diagnostic and prognostic accuracy and sensitivity of miRNA-21 in acute myocardial infarction (AMI) by performing an evidence-based meta-analysis of previous AMI-related clinical studies.

**Methods:**

Chinese and English literature published before April 2024 were searched, and data were reviewed and extracted. After quality appraisal, the STATA 16.0 software was used for the effect size analysis of the various treatments described in the literature.

**Results:**

A total of 14 valid documents were retrieved from 562 studies. The results of the systematic review revealed that for the patients with AMI vs. those without non-AMI, the aggregated odds ratio reached 5.37 (95% confidence interval 3.70–7.04). The general sensitivity and specificity for the circulating miRNA-21 levels in diagnosing AMI were 0.83 and 0.81, respectively.

**Discussion:**

Thus, the meta-analysis of 14 AMI-related clinical trials highlighted that miRNA-21 may serve as a promising biomarker for diagnosing AMI.

## Introduction

Coronary artery disease (CAD) is one of the leading fatal clinical threats around the world (1). The prevalence of myocardial infarction is 4.0% in the United States and 3.1% in the UK (2, 3). Diagnosing acute myocardial infarction (AMI) earlier and predicting the severity of myocardial necrosis as early as possible are hot topics in cardiovascular research. Coronary angiography, being the gold standard for the definitive diagnosis of AMI stenosis, requires strict conditions, as it is an invasive procedure. For the early diagnosis of AMI, guidelines advocate the dynamic monitoring of electrocardiograph (ECG) and markers of myocardial necrosis, such as cardiac troponin I (cTnI) and creatine kinase-MB (CK-MB), which are highly precise and specific (1–3).

miRNAs are a highly conserved subset of small noncoding RNAs (4); they play an integral role in suppressing the inflammatory reactivity of atherosclerotic plaques, improving endothelial cell function and cardiac metabolism, alleviating ischaemia, and other pathophysiological activities. Especially, miRNA-21 is considered to be closely related to myocardial injury (4–6).

In ischaemia–reperfusion injury, knockdown of miRNA-21 in mice exacerbates aldosterone-mediated cardiac hypertrophy and injury (7–9). In reperfused myocardium and during post-infarction remodelling, miRNA-21 protects myocytes from ischaemia/reperfusion-induced apoptosis. Moreover, miRNA-21 levels were found to be elevated in tissues post-infarction and miRNA-21 was confirmed to promote collagen fibril secretion by fibroblasts, which not only specifically regulates the repair of vascular injury, but also effectively regulates myocyte metabolic activity, which in turn, affects the prognosis of patients with AMI (10, 11).

Several investigations have revealed that miRNA-21 is expressed at high circulatory levels in CAD patients and that its levels are correlated with the degree of vascular stenosis (12). Meanwhile, the expression of miRNA-21 varies among different types of anginas and myocardial infarction, suggesting that differences in its expression profiles can also predict disease and the stage of disease development. Furthermore, miRNAs are likely to serve as potential therapeutic targets for heart disease (9, 13). Since many randomised controlled trials could not be performed due to diverse differences in disease characteristics, in the present study, we used an evidence-based approach to effectively combine the influence of miRNA-21 expression levels reported in different AMI-based clinical studies and performed a meta-analysis to investigate the accuracy and specificity of miRNA-21 for diagnosing AMI, in comparison with other markers, such as cTnI and CK-MB.

## Methods

### Selection procedures

In accordance with the PRISMA guidelines (14), Pubmed, Embase, and Web of SCI in English and Chinese National Knowledge Infrastructure (CNKI), Wanfang Database in Chinese were searched from the time of their creation until April 2024. Search-term were as follows: (“circulating”or “blood” or “plasma” or “serum”) and (“micro RNA 21” or “miR-21” or “miRNAs”) and (“AMI” or “coronary artery disease” or “acute myocardial infarction” or “schematic heart disease”).

The inclusion criteria for the literature reviewed here are as follows: a. The selected literature must focus on human studies. b. The selected literature must be related to research regarding the circulating miRNA-21 levels and AMI. c. The selected literature must provide adequate records to enable an estimation of the predictive and metabolic status of miRNA-21 in AMI (1).

The exclusion criteria for the literature reviewed here are as follows: a. The literature that does not provide sufficient or applicable data, including case reports, literature reviews, meetings, correspondence letters, editorials, and conference abstracts. b. The articles that are not written in English or Chinese. c. The literature unrelated to AMI. d. The articles published repeatedly, as well as those with small sample size and incomplete information.

### Data extraction and quality-assessment

Two researchers reviewed the titles and abstracts of articles eligible for the review in this study and then extracted the relevant data and information independently. When there were disagreements, they were resolved via discussions between the two researchers or with assistance from a third staff member. Based on the previously mentioned inclusion and exclusion criteria, 14 articles (15–28) were identified for a full-text review. The extracted research records derived included information on original sponsor, years and issues, cases numbers, controls, the determination of AMI, the type of specimen and blood sampling time required for diagnosis, the sensitivity and specificity ratings, the AUC values, and the miRNA-21-detection methods. The risk of bias and applicability concerns were checked using QUADAS-2. The process includes the below four critical fields: patient selection, index detection, reference standards, and the process and timeline. Risk of bias was assessed for each domain and applicability was assessed for the first three domains in Revaman 5.3 software. Researches were classified as being at low, high or unclear risk.

### Statistical analysis

A meta-analysis summarising the research findings was performed. Meanwhile, more than three papers were scrutinised to forecast the predictive and metabolic significance of miRNA-21 levels for diagnosing AMI. The STATA 16.0 software was utilised to perform the data analyses. To calculate the ensemble the collective odds ratios, along with the 95% confidence interval, via fixed or random-effects models, we used the DerSimonian-Laird (D-L) or Inverse-Variance (I-V) statistical approaches. If the heterogeneity test result is *P* > 0.10, multiple studies were homogeneous, as fixed effects model of Inverse-Variance (I-V) could be selected. When *P* < 0.10, inclusive studies were checked for heterogeneity using the D-L method, and analyzed sources of heterogeneity by sub-group. For assessing the accuracy of miRNA-21 in diagnosing AMI, a summary of the sensitivity, specificity, positive likelihood ratio (PLR), and negative likelihood ratio (NLR), and diagnostic odds ratio (DOR) were calculated,which indicated the accuracy of microRNAs in the differentiation of AMI and no AMI, were calculated from the TP, FP, FN, and TN. For *I^2 ^*> 50% or *p *< 0.05, the bivariate summary receiver operator characteristic (SROC) curve showed a downward trend, after which the AUC could be combined to ascertain the accuracy of miRNA-21 for diagnosing AMI, relative to the controls.

## Results

### Literature search results

**Figure 1:**
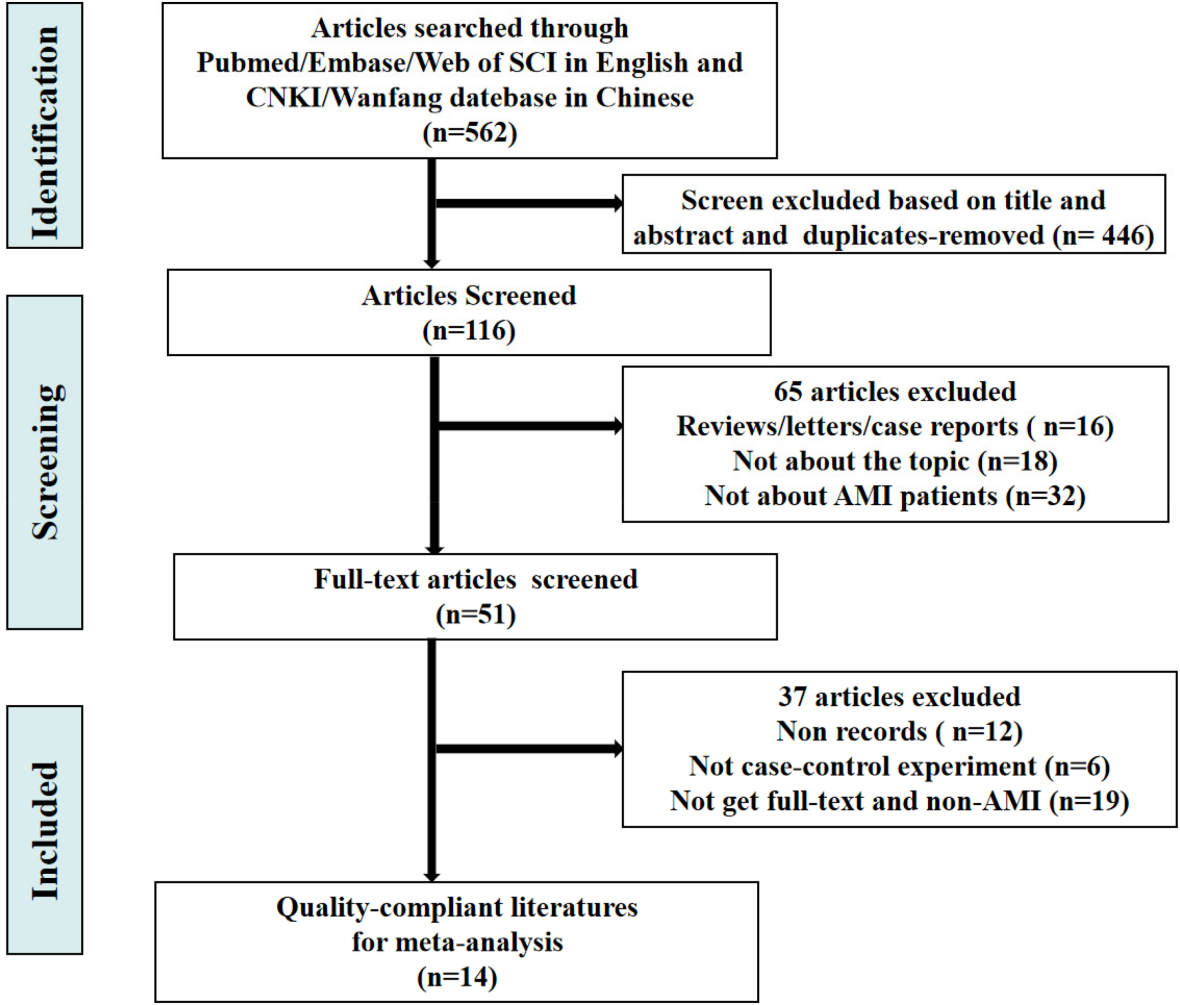
Flowchart of the selection process and cases inclusion.

**Figure 2:**
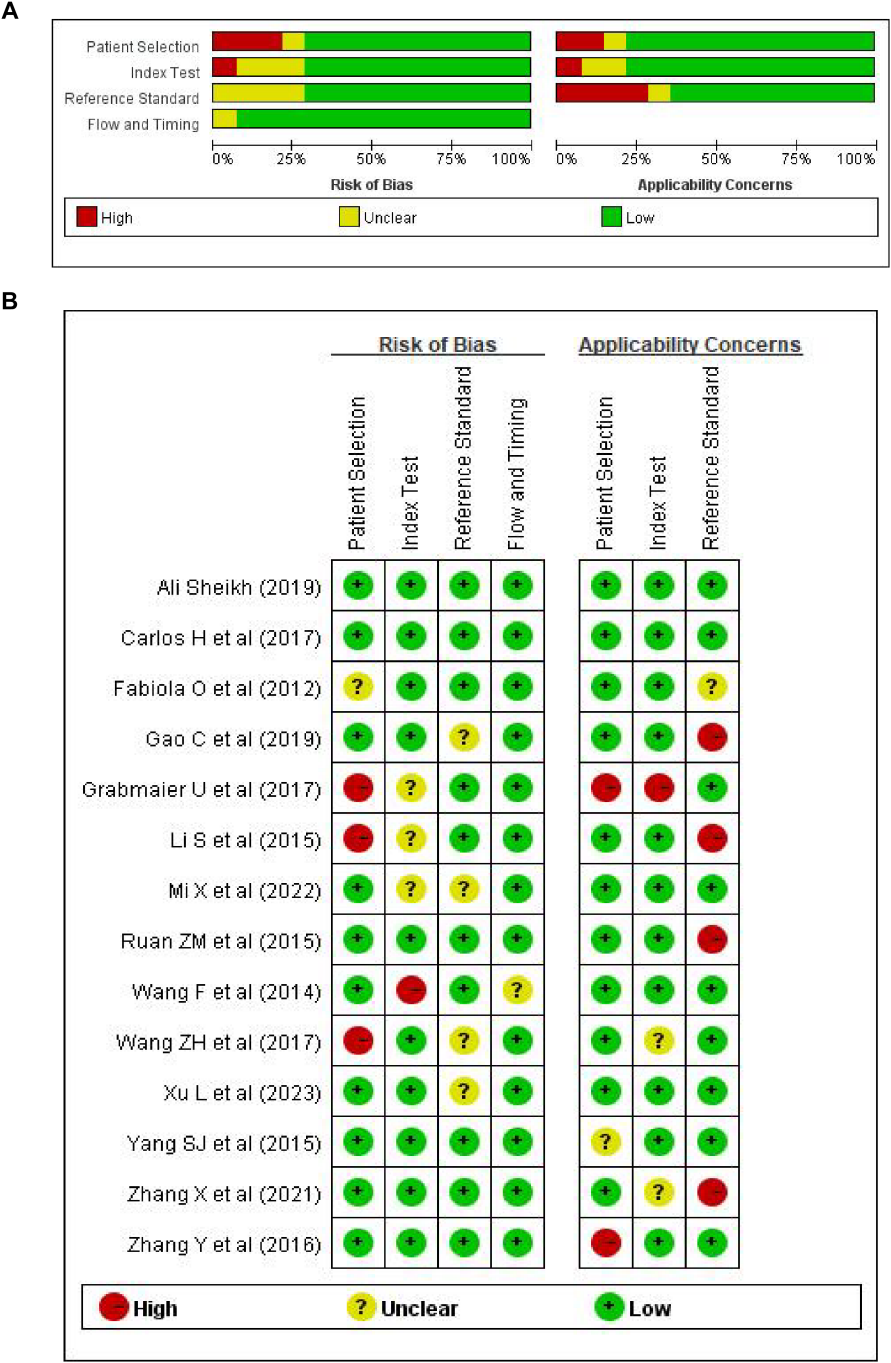
Risk of bias graph. **(A)** Review authors’ assessment of each risk of bias as percentage of all included studies; **(B)** risk of bias summary.

The retrieval process of original literature is presented in Figure 1. Based on the criteria for literature inclusion and exclusion, we got 562 records for miRNA-21, with 165 in duplicates -removed and 281 not for AMI clinical experiments or be without no full test then removed after title and abstract reading (*n* = 446), 116 articles were screened in full-text in English and Chinese publications regarding miRNA-21. In the end, 14 articles were included (14–25), the basic information of which is shown in Table 1. The risk bias was calculated using the Cochrane bias-risk tool and is presented in Figure 2.

**Table 1:** Characteristics of the 12 studies included in our meta-analysis.

Study	Country	Study design	Specimen	Case (*n*)	Control (*n*)	Methods	Diagnosis Reference standard	Time for detection	AUC	Sensitivity	Specificity
Zhang X et al. (15)	China	Retrospective Clinical Study	Serum	24 (AMI)	27 (Non-AMI)	SYBR	MACE, PCI	Within 24 h	0.824	83.90%	87.60%
Yang SJ et al. (16)	China	Retrospective Clinical Study	Plasma	17 (AMI)	10 (healthy)	SYBR	cTnI, CK-MB	Within 12 h	0.6885	73.76%	78.47%
Zhang Y et al. (17)	China	Prospective Cohort Study	Plasma	17 (AMI)	10 (Non-AMI)	TaqMan	CK, cTnI, CK-MB	0–3, 3–6, 6–9, 9–12, and 12–24 h	0.892	86%	81%
Grabmaier U et al. (18)	Austria	Prospective Cohort Study	Plasma	44 (AMI)	18 (Healthy)	TaqMan	ΔIV, ΔLVEF, ΔLVEDV	Within 12 h	NR	NR	NR
Wang ZH et al. (19)	China	Clinical Study	Serum	38 (NSTAMI)	25 (Healthy)	SYBR	cTnI, CK-MB	Within 12 h	0.981	97%	80%
Ali Sheikh (20)	China	Clinical Study	Serum	123 (SA)/82(UA)	50 (Healthy)	SYBR	AHA, cTnI, CK-MB	NR	0.921	83%	95%
Li S et al. (21)	China	Clinical Research	PBMCs	24 (AMI)	27 (Non-AMI)	SYBR	AHA	Within 24 h	NR	NR	NR
Wang F et al. (22)	China	Prospective Cohort Study	Plasma	17 (AMI)	28 (Healthy)	SYBR	cTnI	Within 0 h, 4 h, 12 h, 24 h, 48 h, 72 h.	0.949	NR	NR
Fabiola O et al. (23)	Italy	Retrospective Cohort Study	Plasma	92 (AMI)	99 (Healthy)	SYBR	cTnT	Within 48 h, 72 h	0.86	NR	NR
Carlos H et al. (24)	Spain	Prospective Cohort Study	Serum	40 (NSTAMI)	20 (Healthy)	TaqMan	NR	NR	NR	NR	NR
Gao C et al. (25)	China	Retrospective Cohort Study	Plasma	184 (AMI)	150 (Healthy)	SYBR	cTn	Within 6 h	0.617	80.00%	40.94%
Ruan ZM et al. (26)	China	Retrospective Cohort Study	PBMCs	22 (AMI)	32 (Non-AMI)	SYBR	PCI	Within 24 h	NR	NR	NR
Mi X et al. (27)	China	Original Research	Plasma	40 (AMI)	44 (Healthy)	SYBR	cTnI, CK-MB	Within 12 h	0.98	NR	NR
Xu L et al. (28)	China	Original Research	Plasma	51 (AMI)	50 (Healthy)	SYBR	cTnI, CK-MB	Within 6 h, test in 3 months	0.66	82.50%	45.50%

AHA, American Heart Association; AMI, acute myocardial infarction; SA, stable angina; UA, unstable angina; NSTAMI, non-ST elevation myocardial infarction; SYBR, SYBR Green I; MACE, major adverse cardiovascular events; ΔIV, ΔInfarct volume; ΔLVEF, ΔLeft ventricular ejection fraction; ΔLVEDV, ΔLeft ventricular end-diastolic volume; NR, no report; CK-MB, Creatine Kinase MB isoenzyme; cTnI, cardiac troponin I; cTnT, cardiac troponin T; cTn, cardiac troponin.

There included 1,405 patients in this article, as 815 AMI patients and 590 control groups (494 healthy controls, 96 non-AMI patients). Three kinds of specimen (Serum, Plasma, peripheral blood mononuclear cells-PBMCs) were in inspection in 14 records. MiRNA-21 test methods were SYBR Green for PCR in 11 articles and TaqMan MicroRNA Assays in 3 articles. 11 articles for patients in China and 3 in Austria/Italy/Spain.3 articles in Chinese languages and 11 in English.

### The diagnostic accuracy of miRNA-21

**Figure 3:**
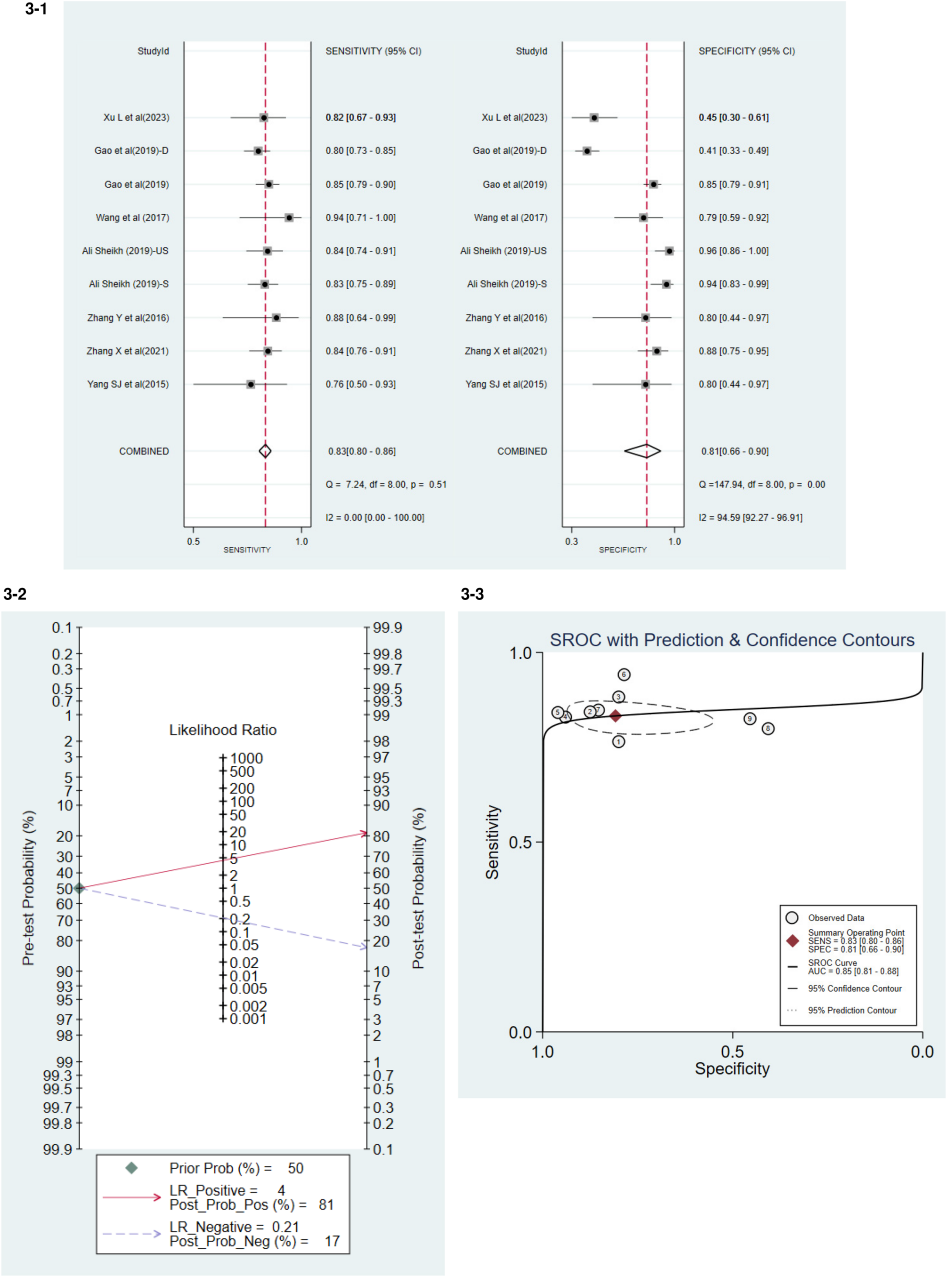
**3-1** Accuracy of miRNA-21 for diagnosing AMI. **3-2** Positive and Negative Likelihood Ratio. **3-3** Summary Receiver Operator Curve (SROC).

Seven studies have detailed the sensitivity and specificity of miRNA-21 in the diagnosis of AMI, among which the paper by Ali Sheikh et al. (2019) categorized the population into Stable Myocardial Infarction (S) and Unstable Myocardial Infarction (US), while the study by Gao et al. (2019) further identified a sub-group of Deceased (D). The accuracy of miRNA-21 for diagnosing AMI was assessed (Figure 3-1). The levels of circulating miRNA-21 demonstrated an overall sensitivity of 0.83 (95% CI, 0.80–0.86) and a specificity of 0.81 (95% CI: 0.66–0.90).

The Positive Likelihood Ratio (PLR) was 4, and the Negative Likelihood Ratio (NLR) was 0.21 (Figure 3-2). PLR was the ratio of true positives to false positives. The higher the ratio, the more likely the test result was a true positive if positive. The smaller the ratio of NLR, the more likely the test, if negative, will be true negative.

The combined weighted area under the Summary Receiver Operating Characteristic curve (SROC) was 0.85 (95% CI, 0.81–0.88) (Figure 3-3).

### Publication bias

**Figure 4:**
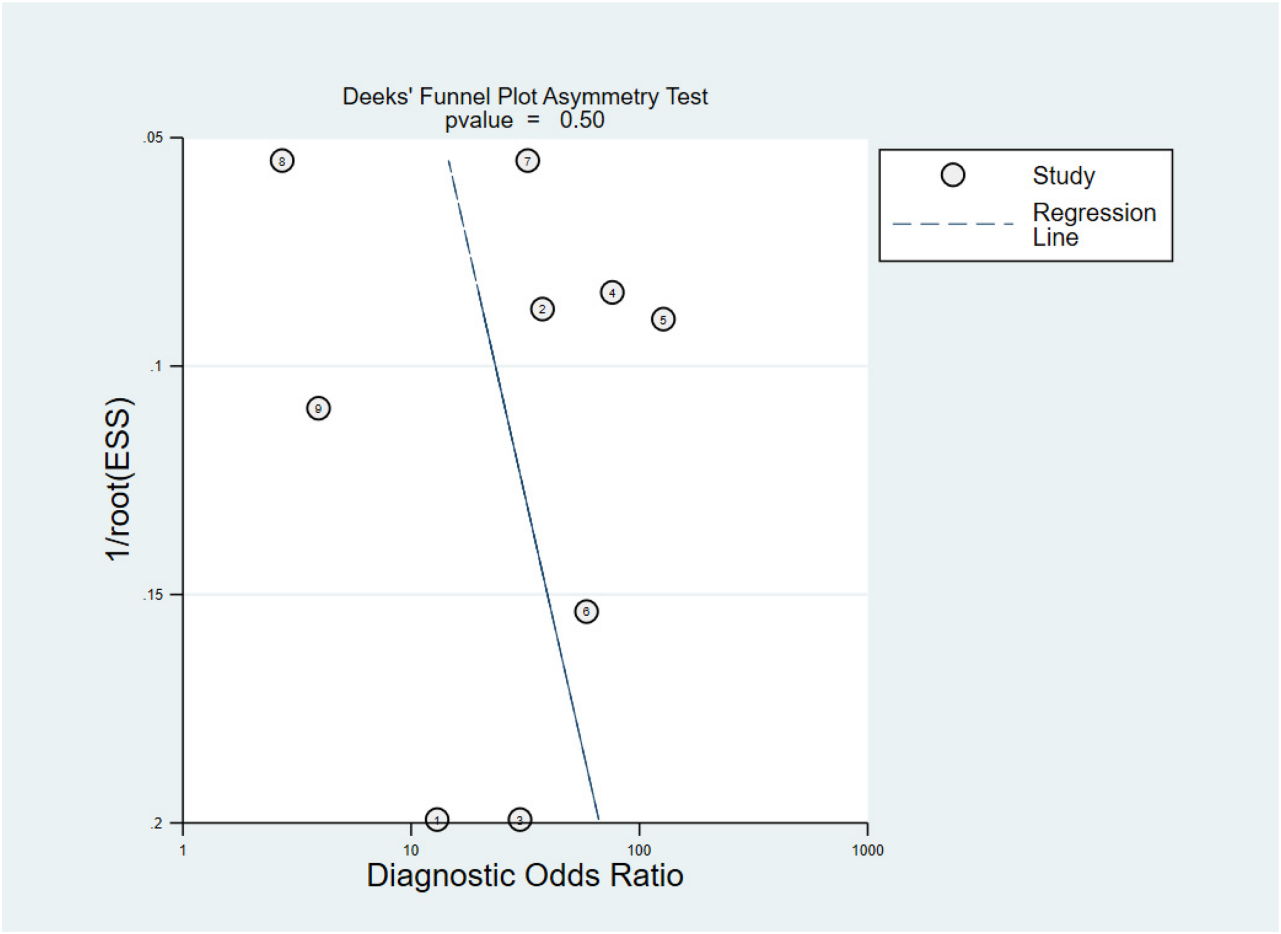
Deeks’ funnel plot asymmetry test.

To examine the potential publication bias, Deeks’ Funnel Plot Asymmetry Test was performed. *P* value < 0.10 were used to judge asymmetry when the number of studies was small. The *p*-value was found to be 0.50, which implies that the publication bias was moderate (Figure 4).

### Summary of findings

**Figure 5:**
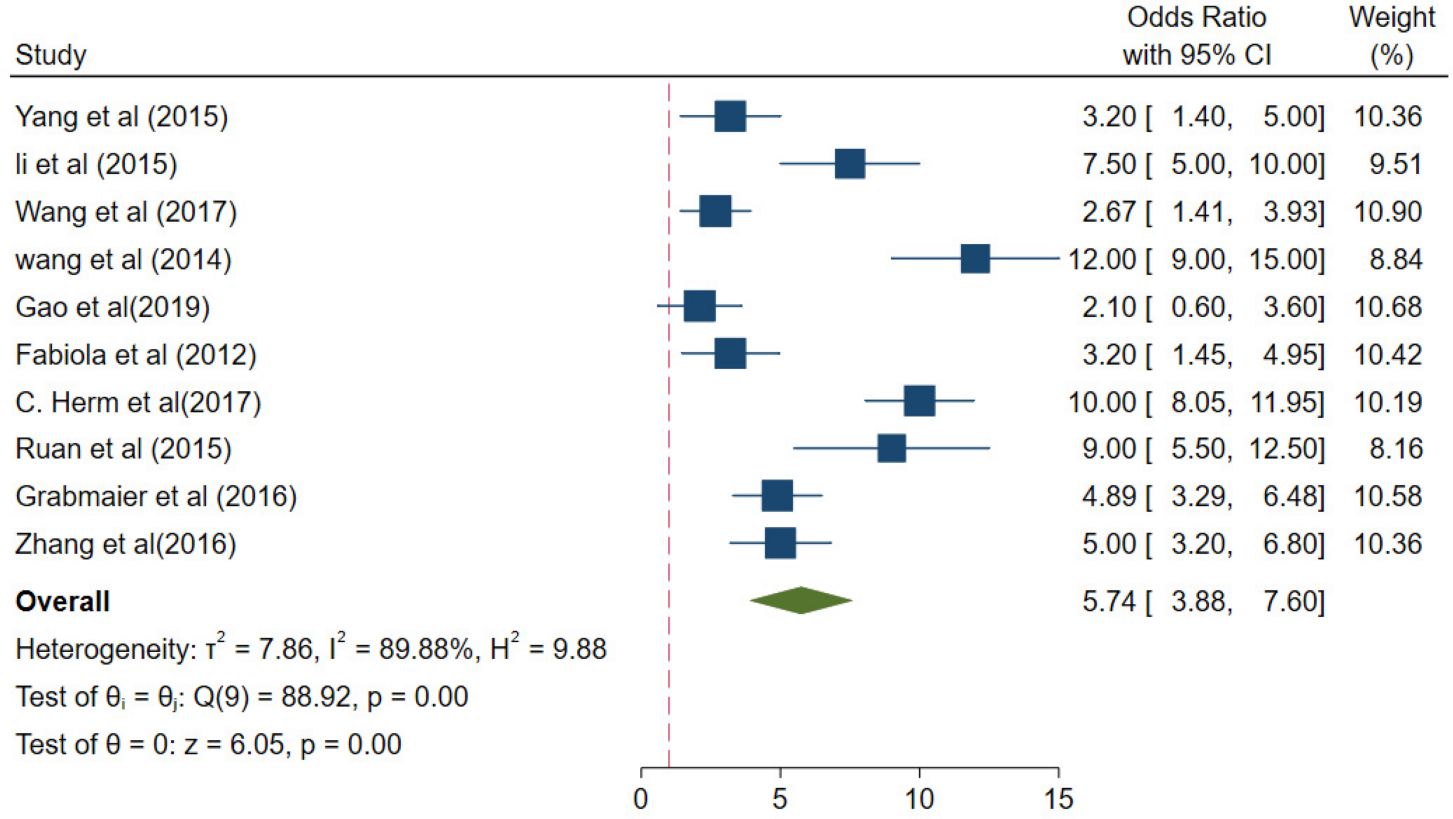
The collective odds ratio (OR) of the miRNA-21 levels.

In the patient group, the collective odds ratio (OR) of the miRNA-21 levels was 5.74 (95% CI 3.88–7.60), compared with the control group (Figure 5). The *I*^2^ value was 89.88%, and the *p*-value < 0.01; there was a high degree of heterogeneity in the results from different studies. Therefore, the sources of heterogeneity were investigated through subgroup analysis.

### Results of the subgroup meta-analysis

**Figure 6:**
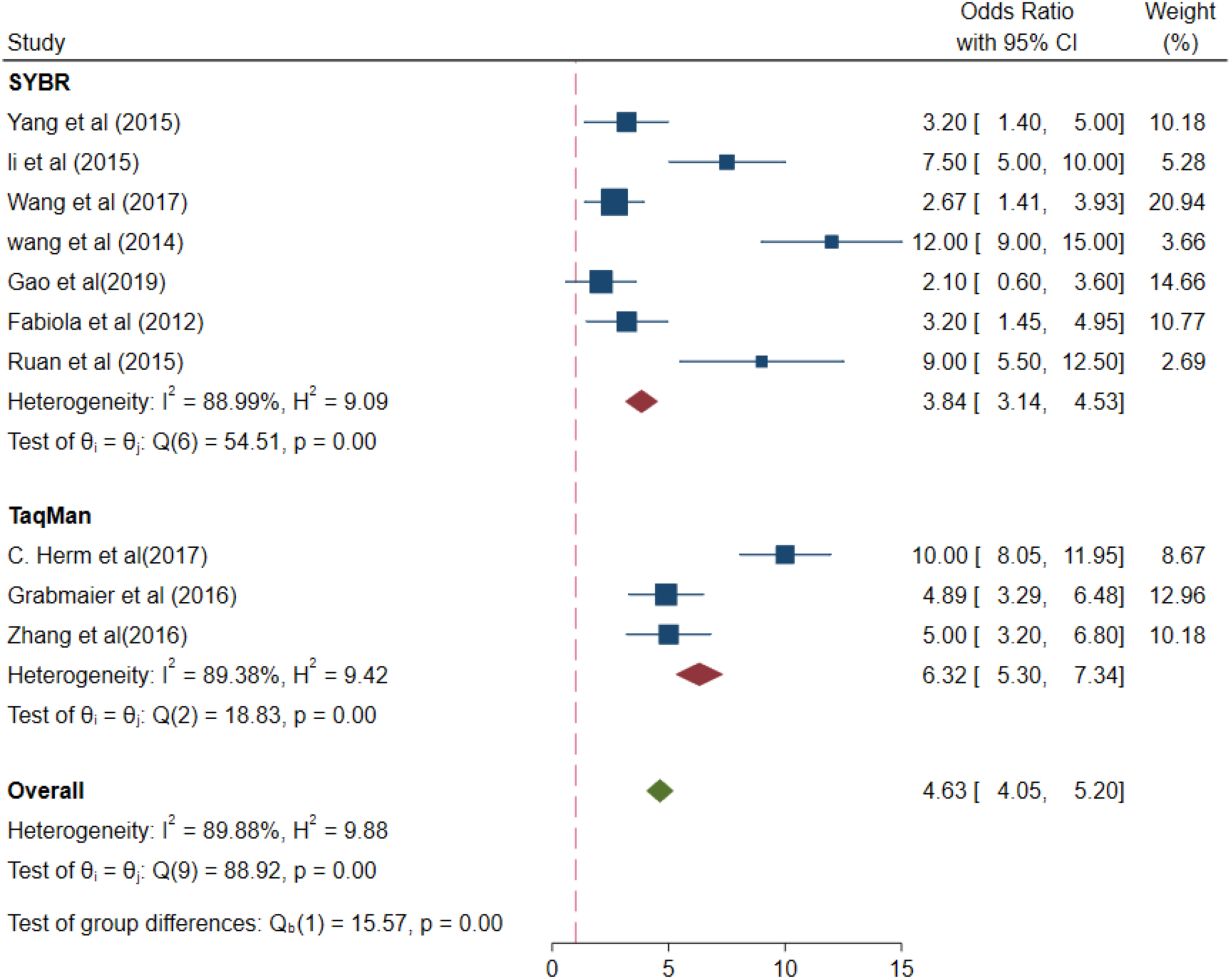
Subgroup analysis –SYBR and taqman (different detection methods).

A subgroup meta-analysis was performed based on the outcomes of the meta-regression analysis. The values of different parameters for the miRNA-21 levels measured using different detection methods (SYBR and Taqman assays) were for the SYBR method (OR 3.84, 95% CI 3.14–4.53) and *I*^2 ^= 88.99% (*p* < 0.01), with for the Taqman method (OR 6.32, 95% CI 5.30–7.34) and *I*^2 ^= 89.38% (*p* < 0.01) (Figure 6). Taqman was preferable to SYBR.

**Figure 7:**
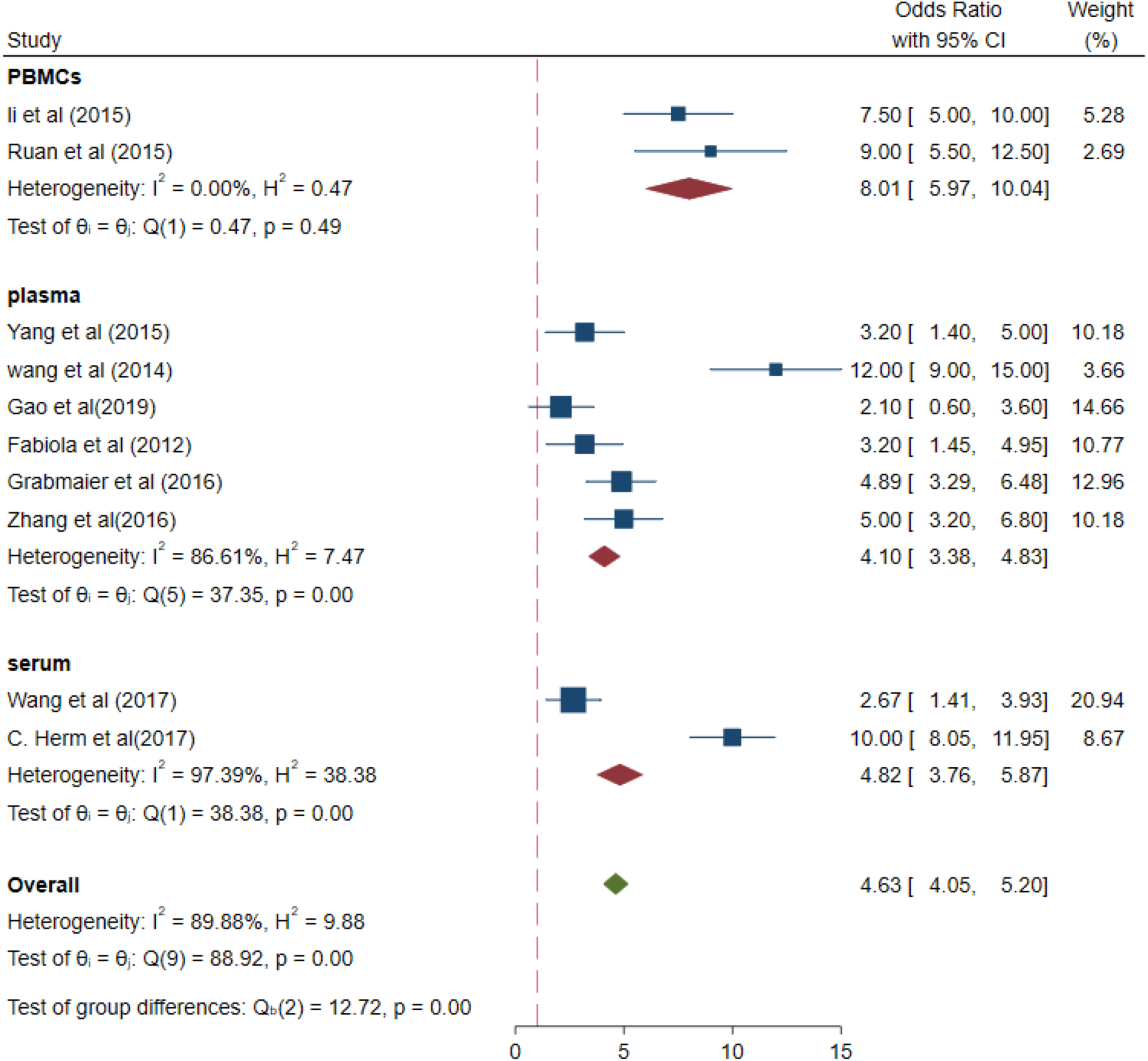
Subgroup analysis –plasma, PBMCs and serum (different samples).

The values of different parameters for the miRNA-21 expression levels in samples were for PBMCs samples (OR 8.01, 95% CI 5.97–10.04), *I*^2^ = 0%, *p *= 0.49 > 0.01; for plasma (OR 4.10, 95% CI 3.38–4.83), *I*^2^ = 86.61% (*p* < 0.01); and for serum samples (OR, 4.82, 95% CI 3.76–5.87), *I*^2^ = 97.39% (*p* < 0.01; Figure 7). Increased- heterogeneity arises from different assays.

**Figure 8:**
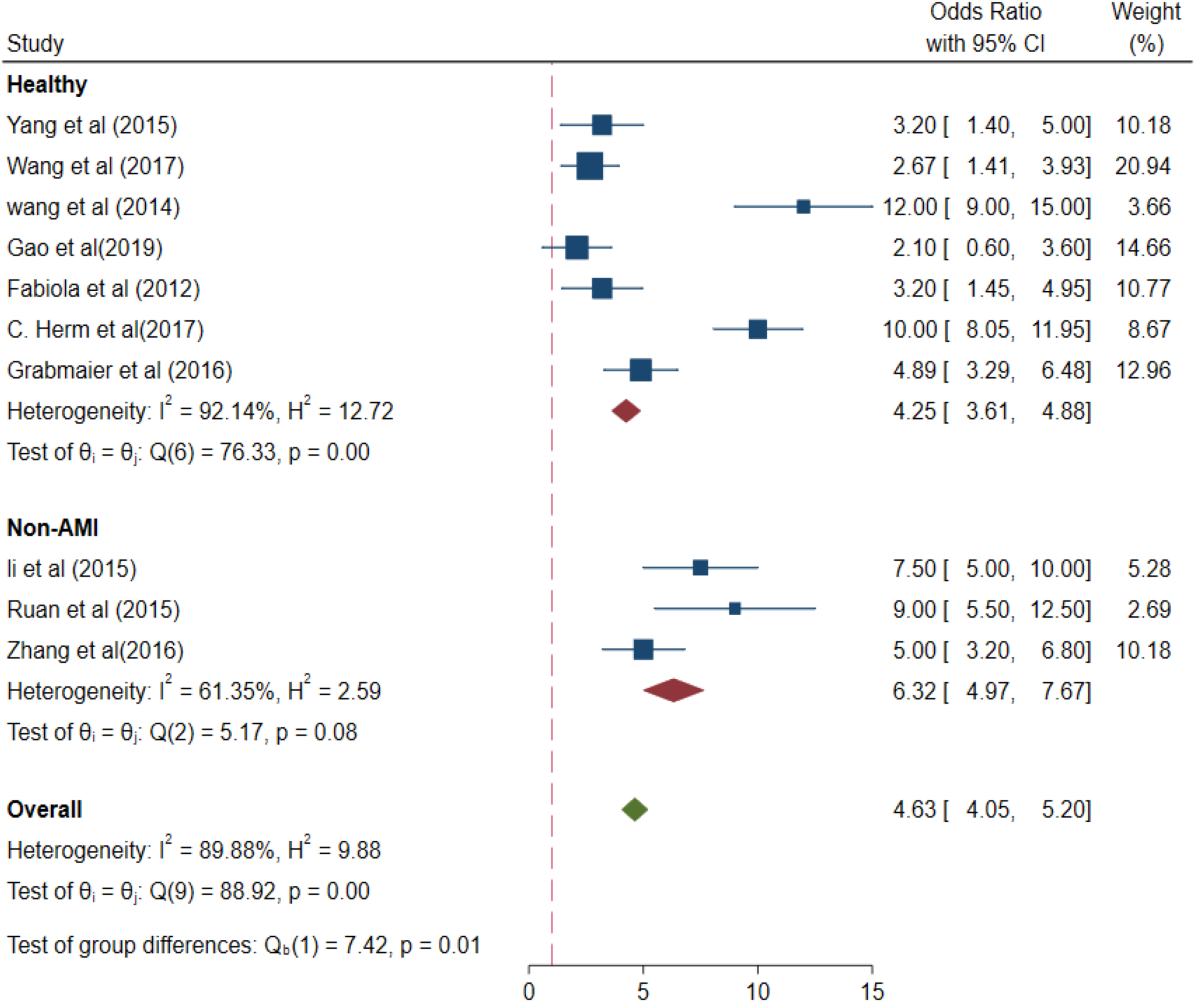
Subgroup analysis –healthy and Non-AMI (different control groups).

The values of different parameters for the miRNA-21 expression levels in the control group based on the selection differences were as follows: healthy control group (OR 4.25, 95% CI 3.61–4.88) and non-AMI group for control (pooled OR 6.32, 95% CI 4.97–7.67), *p* < 0.01 (Figure 8).

**Figure 9:**
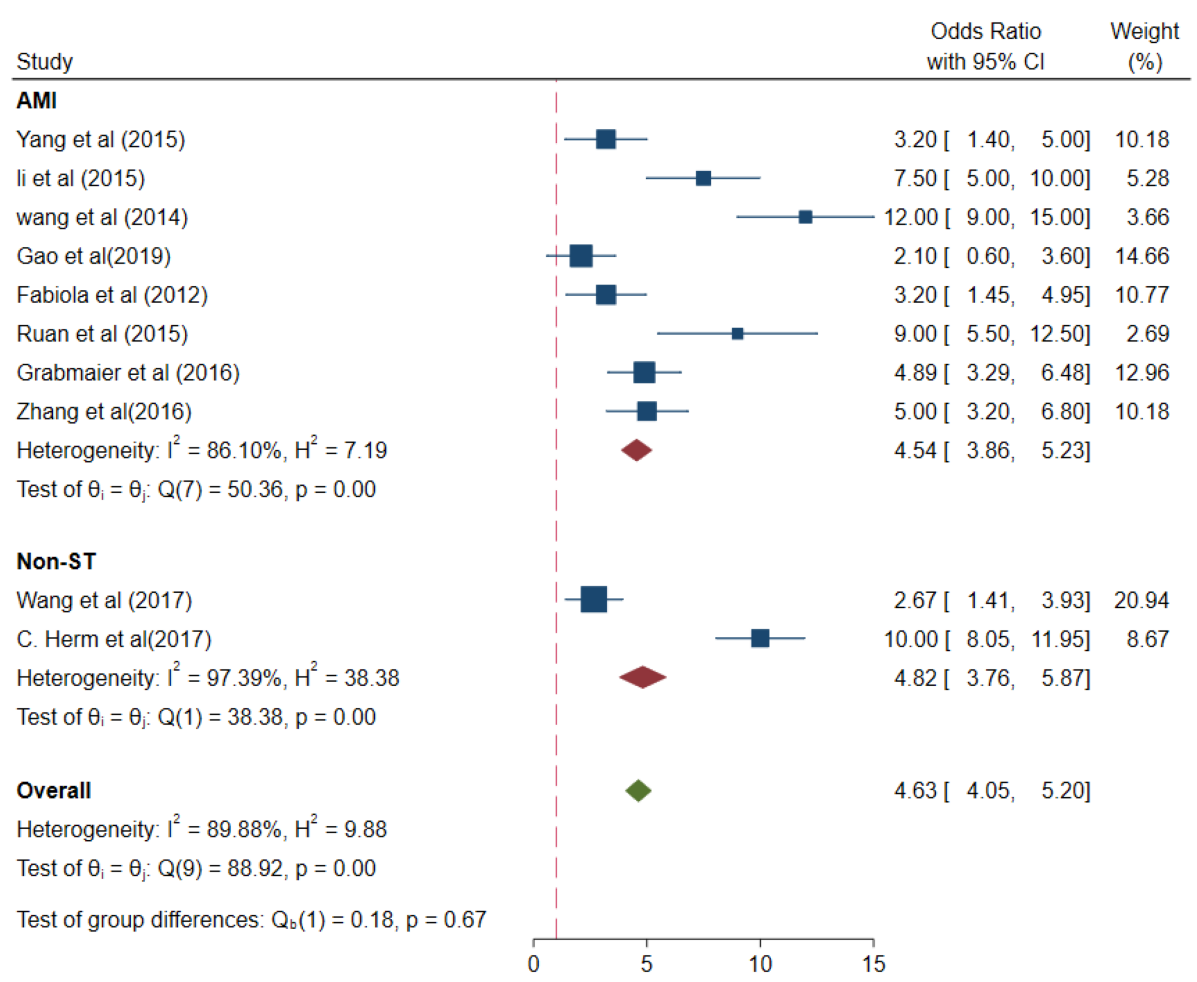
Subgroup analysis –AMI and NSTAMI (different case groups).

The values of different parameters for the miRNA-21 expression levels in the case group were as follows: AMI cases, (pooled OR 4.54, 95% CI 3.86–5.23); *I*^2^ = 86.1%; and *p* < 0.01 and non-ST-elevation myocardial infarction (NSTEMI) cases, (pooled OR 4.82, 95% CI 3.76–5.87); *I*^2^* *= 97.39%; and *p* < 0.01 (Figure 9).

**Figure 10:**
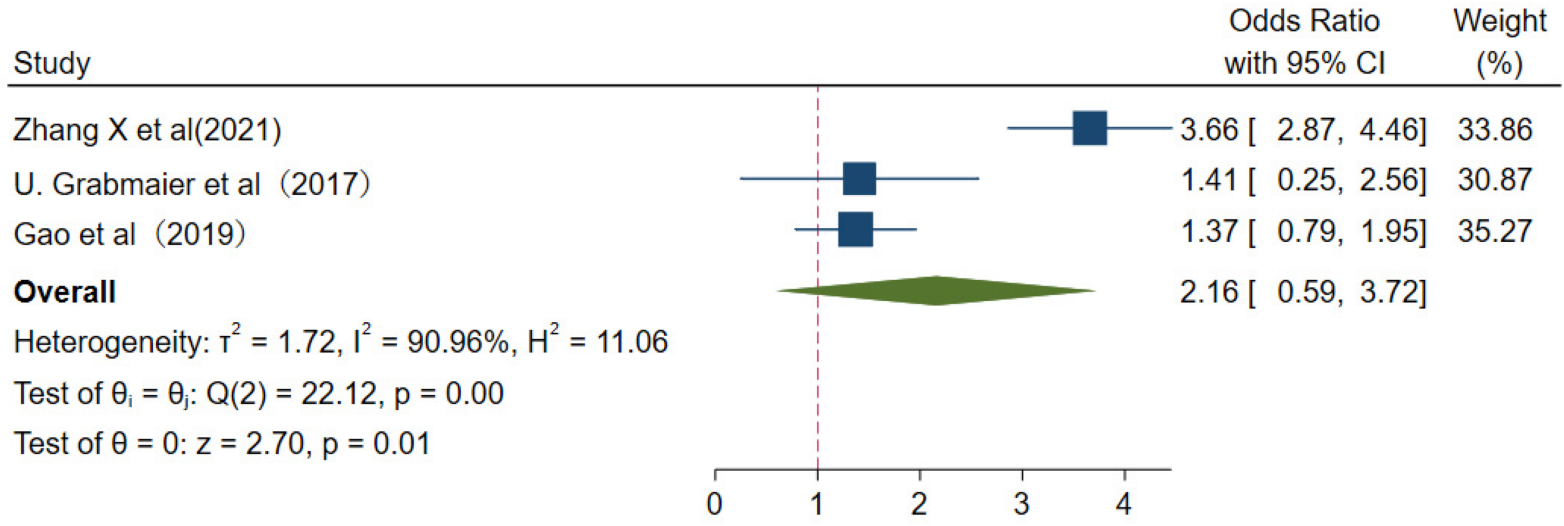
Major adverse cardiovascular events.

From major adverse cardiovascular events, the values were pooled OR 2.61 (95% CI 0.59–3.72); *I*^2^ = 90.96%; and *p* < 0.01 (Figure 10).

## Discussion

Studies have revealed that miRNA-21 has strong sensitivity and specificity as a predictive and diagnostic indicator for AMI. In serum and plasma, the miRNA-21 levels were remarkably elevated, compared with those in the controls. A serious cardiovascular disease, AMI shows a rapid onset and a high risk of mortality (29, 30). Thus, the early diagnosis and treatment of AMI has become an urgent need in recent years.

In the present study, the area under the curve (AUC) showed that miRNA-21 may serve as a reliable diagnostic indicator for AMI. A PLR of 4 implies that a person with AMI is fourth times more likely to have a positive diagnosis than other patients. The NLR indicates that a person with AMI has 21% less chance of diagnosis if miRNA-21 is negative. These results strongly suggest that circulating miRNA-21 is a potential predictor.

As the generally recognized gold standard, circulation cTnI could be found until 3–6 h after AMI, CK-MB could be detected until 6 h when AMI (31). There were constraint in on-time predictive and diagnostic functions. Furthermore, chronic kidney diseases could also be along with cTnI raising-up (32), which desperate to seek for sensitive and highly-accuracy biomarker. MiRNA-21 could be detected within three hours in AMI patients, what's more, it's effective in assessing the degree of myocardial ischemia (31). Combinations with ECG and other biomarkers could be in consistent further studies (31, 32).

Different types of methods for the real-time quantification of miRNA-21 levels, i.e., fluorescence methods such as Taqman probe assay and SYBR Green assay revealed that the miRNA-21 levels were 6.32-fold and 3.84-fold higher, respectively, than those in the healthy controls. miRNAs are a small class of homologous molecules; some small miRNAs may be expressed at very low levels, due to which extremely sensitive and quantitative assays are required for their detection, Taqman probe assay and SYBR Green assay were general and highly sensitivity for miRNA-test in clinical laboratories (30). In plasma, and serum, the miRNA-21 levels were found to be 4.10- and 4.82-fold higher, respectively, than those in the control cases. Conversely, in different types of blood samples from AMI patients, the miRNA-21 levels were reported to be increased.

In the analysis of different subgroups relative to the control group, the miRNA-21 level in the AMI group was 4.25-fold higher than that in the healthy group, while compared with the non-AMI group for control, the miRNA-21 concentration was 6.32-fold higher. Thus, the circulating miRNA-21 level is a good diagnostic indicator for AMI in healthy individuals and those with a history of CAD. With regard to the experimental group, the miRNA-21 levels of unclassified AMI patients were 4.54-fold higher than those in the controls. The miRNA-21 levels in patients with NSTEMI miRNA-21 were 4.82-fold higher than those in the controls, implying that miRNA-21 is a diagnostic indicator that is very closely associated with NSTEMI.

Numerous studies (2, 15, 33, 34)^,^ have shown that plasma miRNA-21 levels can effectively assess the severity of ischaemia and show a high predictive accuracy for the prognosis of adverse cardiovascular events at different periods. Zhang X (15) found plasma miRNA-21 levels were higher in AMI patients who developed MACE at 1-year follow-up. In the study of Mi (27), miRNA-21 levels were increased in infarct-related artery total occlusion and or infarct related blood-vessel recanalization patients.Range researches (16, 28) showed it also a good indicator to predict vascular restenosis post-PCI in advance.

The miRNA-21 level has good diagnostic value for analysing AMI patients who received early revascularisation therapy. In addition, miRNA-21 exerts protective effects on ischaemia-reperfused myocardium. Yin (9) performed heat shock pretreatment in a mouse model *in vivo*, and found that several miRNAs, including miRNA-21, could reduce the infarct size. Weber (10) found that reducing the degree of apoptosis via miRNA-21 over-expression improved endothelial function and slowed down the progression of atherosclerosis. A high level of miR-21 expression may promote inflammatory aggregation, induce apoptosis of cardiomyocytes and worsen the progression of myocardial fibrosis,making it the best target for specific diagnosis and treatment of CVD (35).

As a biomarker in the early stage of AMI, miRNA 21 may be superior to cardiac troponin (cTn) I. miRNA 21 could be detected in the plasma of all patients within 4 h of symptom onset, as cTnI detected in only 85% patients. The ECG parameters were combined with the peripheral blood miR-21 provide a reference for the clinical diagnosis of Acute Myocardial Infarction (18, 28). There is an urgent need for more clinical studies on the diagnostic efficacy of combined tests.

Despite the small bias and strong sensitivity and specificity, this study has a few limitations: (1) there is a gap with regard to the results of evidence-based clinical studies and randomised controlled trials; (2) the time of blood collection was not uniform in various studies; and (3) the inclusion criteria for the diagnosis of AMI in clinical trials were not uniform, since some used percutaneous coronary intervention to determine the degree of stenosis and others used the levels of cardiac markers.

## Conclusions and future perspectives

This study underscores that circulating the miRNA-21 level shows great potential possibility to serve as a reliable biomarker for the clinical diagnosis of AMI. In addition to the diagnostic gold standards for AMI diagnosis, i.e., the levels of cTnI, CK-MB, and other cardiac enzymes, to confirm the utility of miRNA-21 as a diagnostic indicator of AMI, we need large scale and rigorous clinical trials to validate the changes in the circulating levels of miRNA-21 in AMI patients and explore the specific biomedical mechanisms underlying these changes. Furthermore, whether the levels of different miRNAs can be combined or form a combined signature for increasing the diagnostic accuracy of AMI is yet to be ascertained.
